# Cell-Type Specific Changes in Glial Morphology and Glucocorticoid Expression During Stress and Aging in the Medial Prefrontal Cortex

**DOI:** 10.3389/fnagi.2018.00146

**Published:** 2018-05-23

**Authors:** Thomas E. Chan, Yael S. Grossman, Erik B. Bloss, William G. Janssen, Wendy Lou, Bruce S. McEwen, Dani Dumitriu, John H. Morrison

**Affiliations:** ^1^Department of Neuroscience, The Friedman Brain Institute, Mount Sinai School of Medicine, New York, NY, United States; ^2^Janelia Research Campus, Howard Hughes Medical Institute, Ashburn, VA, United States; ^3^Dalla Lana School of Public Health, University of Toronto, Toronto, ON, Canada; ^4^Laboratory of Neuroendocrinology, Department of Neuroscience, Rockefeller University, New York, NY, United States; ^5^California National Primate Research Center, Department of Neurology, University of California, Davis, Davis, CA, United States

**Keywords:** microglia, glia, glucocorticoids, prefrontal cortex, aging, chronic stress, stress

## Abstract

Repeated exposure to stressors is known to produce large-scale remodeling of neurons within the prefrontal cortex (PFC). Recent work suggests stress-related forms of structural plasticity can interact with aging to drive distinct patterns of pyramidal cell morphological changes. However, little is known about how other cellular components within PFC might be affected by these challenges. Here, we examined the effects of stress exposure and aging on medial prefrontal cortical glial subpopulations. Interestingly, we found no changes in glial morphology with stress exposure but a profound morphological change with aging. Furthermore, we found an upregulation of non-nuclear glucocorticoid receptors (GR) with aging, while nuclear levels remained largely unaffected. Both changes are selective for microglia, with no stress or aging effect found in astrocytes. Lastly, we show that the changes found within microglia inversely correlated with the density of dendritic spines on layer III pyramidal cells. These findings suggest microglia play a selective role in synaptic health within the aging brain.

## Introduction

The prefrontal cortex (PFC) is responsible for the flexible use of goal-directed mental strategies. The prelimbic (PL) area of the rodent medial prefrontal cortex (mPFC) has been suggested to be involved in emotional, cognitive and mnemonic processes (Diorio et al., 1993; Vertes, 2004). The mPFC is highly susceptible to alterations in neural architecture due to exposure to acute and chronic stressors when compared to other regions of the brain. The PL has been shown to have extensive structural changes due to chronic stress exposure, including dendritic length, branching and spine density (McEwen and Morrison, 2013). In addition, similar structural changes have been observed in the PL during aging including age-related spine loss, shifts in spine morphology and postsynaptic density size (Bloss et al., 2011, 2013).

In addition to its role in cognition, the mPFC is part of a neuroendocrine feedback loop regulating the secretion of adrenal hormones in response to stress throughout aging (Diorio et al., 1993; Sapolsky, 1999; McEwen, 2006). Environmental stressors activate the hypothalamic-pituitary-adrenal axis, culminating in the release of glucocorticoids from the adrenal cortex (Treccani et al., 2014). Glucocorticoids act on target cells through cytoplasmic glucocorticoid receptors (GR) that are expressed in various cell types including pyramidal cells, microglia and astrocytes in the central nervous system (Sierra et al., 2008; Yu et al., 2011). Upon binding glucocorticoids, activated GRs can trigger several intracellular signaling pathways in the cytoplasm as well as translocate into the nucleus to act as a transcription factor regulating gene expression (van Steensel et al., 1995; Rhen and Cidlowski, 2005; Du et al., 2009; Treccani et al., 2014). Dysregulation of GR expression in neural networks has been associated with memory loss, psychosis and neurodegenerative models (Landfield et al., 2007; Ros-Bernal et al., 2011; Mika et al., 2012; Sinclair et al., 2013; Givalois, 2014).

Although GR expression in neurons within the PFC has been extensively characterized, little is known of the role of GR expression in other cell types (Mizoguchi et al., 2003; Chiba et al., 2012; Popoli et al., 2012; McEwen and Morrison, 2013). Glial cells play a key role in the maintenance of the neuroinflammatory profile in the brain. Microglia act as native macrophages creating an immune response against plaques, cellular degeneration and infection within the central nervous system (Conde and Streit, 2006; Tremblay et al., 2011). Astrocytes also contribute to the immune response by releasing factors that influence activation of microglia and repair of neuronal damage (Shih et al., 2006). Binding of glucocorticoid to GR is an important step in the activation of a neuroinflammatory response and recruitment of glial cell mediated processes (O’Callaghan et al., 1991; Frank et al., 2012). Interestingly, it has been observed that increased recruitment of microglia and astrocytes occurs with age (Cotrina and Nedergaard, 2002; Sierra et al., 2007). A heightened inflammatory profile results from decreased morphologic complexity and increased basal inflammatory cytokines in microglia (Sierra et al., 2007). Moreover, many studies have used soma size as an indicator of changes in immunoreactivity in microglia, namely, increased activation (Kozlowski and Weimer, 2012; Kongsui et al., 2014; Davis et al., 2017). These changes have been postulated to have long-term implications for synaptic structure and plasticity through synaptic stripping. This event occurs at the tripartite synapse, where glial interactions influence pre and postsynaptic remodeling (Trapp et al., 2007; Kettenmann et al., 2013).

To better understand how stress and aging affect glial networks in the PL area of the mPFC, we examined glial cell morphology. Both astrocytic and microglial structures were imaged using confocal light microscopy to determine morphological changes under a chronic stress paradigm in both young and aged rats. Although no chronic stress effects were observed, a significant aging effect was found in microglia morphology. Furthermore, nuclear and non-nuclear GR expression in glial cells was characterized. Our results revealed no significant nuclear GR differences; however, non-nuclear GRs were up-regulated with age.

## Materials and Methods

### Animals and CRS Paradigm

All animals and behavior reported here are in accordance with methods used in previous studies conducted in our laboratories (Bloss et al., 2010, 2011, 2013). Male Sprague Dawley rats were purchased from Harlan Laboratories and allowed 1 week to acclimate. Animals were single-housed in clear polycarbonate cages (45 × 25 × 20 cm) with woodchip bedding at Rockefeller University (*n* = 20–22 per age group). Rats were 3 months old and 20 months old at time of arrival. Although a middle aged group used in previous studies, it was omitted from the present study in light of data presented in previous articles published using this same tissue. Therefore, this study used animal tissue from two time points designated as young (3 months) and aged (20 months) groups. The rats were exposed to 12 h/12 h light-dark cycles at 21 ± 2°C and fed standard rat chow and water *ad libitum*. The rats were weighed every 3 days throughout the course of the experiment.

Under the CRS paradigm, rats were placed in wire mesh restrainers with rubberized edges from 10:00 h to 16:00 h for 21 consecutive days. During this 6-h period, the animals were administered water freely. Recovery condition animals were given an additional 21 days under the same conditions and vivarium as the acclimation period during week 1. Thus, six distinct groups were used in this study: Young Control (YC), Young Stressed (YS), Young Recovery (YR), Aged Control (AC), Aged Stressed (AS) and Aged Recovery (AR). The experiment was conducted in compliance to the National Institutes of Health Guidelines for the Care and Use of Experimental Animals. This experiment was approved by the Institutional Animal Care and Use Committee at Mount Sinai School of Medicine and Rockefeller University.

### Perfusions and Tissue Processing

The tissue samples used in this study were taken from the same animals reported on previously with respect to neuronal and synaptic alterations (Bloss et al., 2010, 2011, 2013).

The animals were sacrificed according to the standard of the National Institutes of Health Guidelines for the Care and Use of Experimental Animals as soon as behavioral testing concluded. CRS animals were perfused 24 h following the last restraint. Animals were perfused at 4.5 months and 21.5 months old, respectively. Rats were administered 100 mg/kg sodium pentobarbital until fully anesthetized. The rats were transcardially perfused with 4% paraformaldehyde + 0.125% glutaraldehyde (0.1 M phosphate buffer (PB) at pH 7.3). During this process, the descending aorta was clamped and non-fixed adrenal glands were removed and weighed to record any changes in weight due to changes in glucocorticoid concentration. Brains were removed and post fixed for 6 h before brain sectioning. The brains were cut alternating between one 250 μm and two 50 μm coronal sections using a (Leica, VT1000S) vibratome. Thin sections were stored in cryoprotectant (30% distilled water, 30% Glycerol, 30% Ethylene Glycol, 10% 2× PB at −20°C) until Immunohistochemistry (IHC) was performed. Following tissue processing, left and right hemispheres were not marked and thus, the tissue used in this study does not address lateralization.

### Immunohistochemistry

IHC was performed to stain for Glial fibrillary acid protein (anti-GFAP), anti-CD11b/c antibody (anti-OX-42), and GR antibody (anti-GR). Sections were stained with mouse anti-rat OX-42 at 1:500 (Abcam), chicken anti- rat GFAP at 1:2000 (Abcam), rabbit anti-rat GR at 1:500 (Santa Cruz) followed by secondary antibodies Alexa fluor donkey anti-mouse 488 at 1:400 (Life Technologies, Carlsbad, CA, USA), Alexa fluor donkey anti-rabbit 555 at 1:400 (Life Technologies) and Alexa fluor donkey anti-chicken 647 at 1:400 (Millipore, Burlington, MA, USA). Additional sections were used for rat anti-mouse NeuN staining at 1:1000.

**Figure 1:**
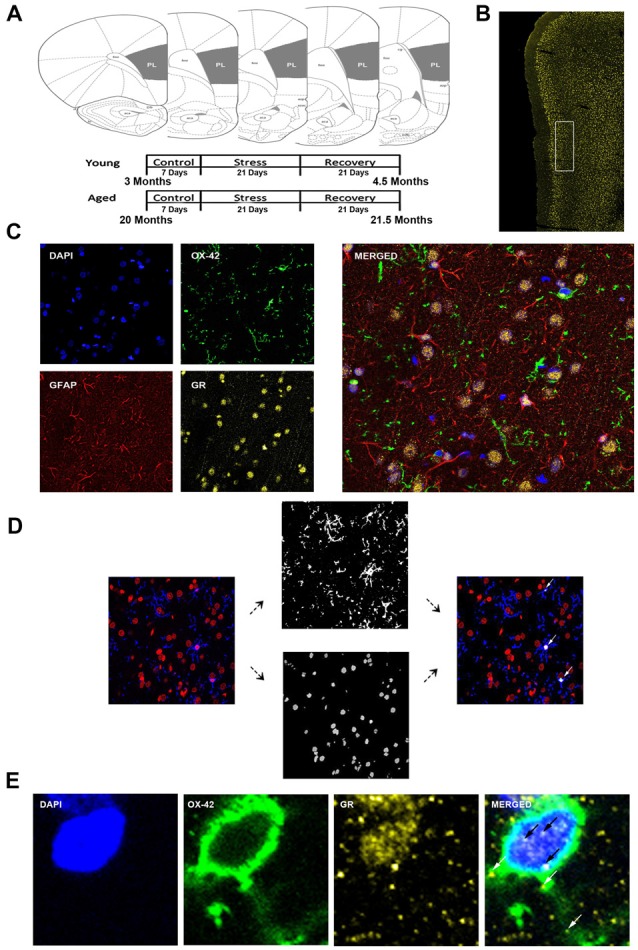
Analysis of medial prefrontal cortical glia. **(A)** Coronal sections of the rat prelimbic (PL) region of the prefrontal cortex (PFC). Young (3 months) and aged (22 months) rats were grouped as control, stress (21 days of 6-h restraint), recovery (21 days post-stress recovery). **(B)** Layer II/III of the PL area outlined on a 50 μm slice. **(C)** Separate channels of DAPI, OX-42, glucocorticoid receptors (GR), and GFAP and a merged immunohistochemical labeling of fixed PFC slices. **(D)** For microglia nuclear analysis, each channel was preprocessed for thresholding to remove noise. OX-42 colocalized DAPI cells were counted using customized colocalization automated method. White arrows indicate microglial nuclei captured by analysis. **(E)** A magnified image of a microglia cell with separate channels of DAPI, OX-42 and GR and a merged immunohistochemical labeling of the PFC. White arrows indicate non-nuclear microglial GR whereas black arrows indicate nuclear microglial GR.

During IHC staining, 50 μm sections were washed in PB solution for 10 min to remove the cryoprotectant. Coronal sections (Bregma +2.20 mm to +4.20 mm) of the PL area of the medial PFC were used for IHC staining (Figure 1A). Sections were first washed in PB once for 5 min and then moved to PB with 0.5% Triton-X detergent for 5 min for four washes. Sections were then moved to a blocking buffer (5% normal donkey serum, 2% Bovine Serum Albumin, 0.2% Cold Water Fish Skin Gelatin, 0.5% Triton detergent in PB) for 1 h on a shaker. Sections were then moved into primary antibody-blocking buffer solution and allowed to incubate on a shaker at 4°C for 24 h. Sections were then washed for 5 min in PB with 0.5% Triton detergent five times. Sections were moved into secondary antibody-blocking buffer solution and allowed to incubate on a shaker at room temperature for 1 h, followed by two washes for 5 min in PB with 0.5% Triton-X detergent. Sections were washed an additional three times for 5 min in PB.

Sections were washed in PB with saline, mounted on Superfrost plus microscope slides (Fisher) and Vectashield for fluorescence with DAPI mounting medium (Vector Laboratories Inc., Burlingame, CA, USA) was applied to the slides before cover slipped (Gold Seal). Slides were stored at 4°C until imaging.

### Confocal Microscopy and Tissue Imaging

The analysis focused on layers II/III of the PL. The correct coronal slices containing PL were determined using landmarks including the rhinal fissure, rhinal incisure, forceps minor and the claustrum and dorsal to the infralimbic area. Layer II/III was determined using DAPI for cell body layer distinction (Figure 1B).

All slides were imaged using a confocal microscope (Zeiss LSM 780) equipped with an Argon laser and ZEN Imaging software. Parameters such as laser excitation and emission channels, pinhole size (one airy disc at 647 nm), frame averaging (eight frames/z-step) were held constant throughout the study. Confocal z-stacks of layer II/III of the PL were taken under oil-immersion using a 1.4 NA plan-Apochromat 40× objective, z-step of 0.5 μm, and a 212.34 μm × 212.34 μm resolution. Four-channel imaging was performed for the entire experiment: DAPI (410–513 nm), OX-42 (491–606 nm), GR (562–660 nm), GFAP (638–755 nm; Figure 1C).

### Image Analysis

Volume, number, intensity of each fluorescent profile and colocalization were extracted using custom-built Javascript and Matlab algorithm. Results were then exported for statistical analysis.

All data analysis was performed on an image-by-image basis. First, the images were imported into Fiji (Schindelin et al., 2012), and separated into the four imaged channels (DAPI, GR, GFAP, OX-42). The intensity distribution of each channel was used to develop a quantitative threshold for excluding noise from the analysis. After thresholding the channels for OX-42 and GFAP, the microglial and astrocytic volumes were collected using Fiji’s 3D image suite and imported into Matlab (Matlab, 2014a). After thresholding the channel for DAPI, images underwent 3D watershed image rendering and were imported into Matlab for individual nucleus identification. The GR location, size, and brightness were collected using Fiji’s Foci Picker 3D (Du et al., 2011), then imported into Matlab for colocalization analysis.

Custom Matlab algorithm was designed to automate identification of different glial subtypes and percentage of nuclear and cytoplasmic colocalization with GR. First, the algorithm identified individual nuclei from the processed DAPI image. It then determined the size of each nucleus and only retained those with a volume between 1000 voxels and 1500 voxels. The retained nuclei were then used to extract voxel information from processed OX-42 images. Only nuclei that completely overlapped (100% colocalization) with OX-42 voxels were identified as microglia (Figure 1D). Once cell type was determined, percent of GR nuclear and cytoplasmic colocalization was computed for the cell (Figure 1E). Our colocalization analysis demonstrated that the majority of nuclei exhibited dense GR staining. To introduce a quantifiable method to this observation, the GR percentage of nuclear volume was divided into quartiles: Q1 = 0%–25% of the nucleus colocalized with GR, Q2 = 25%–50% of the nucleus colocalized with GR, Q3 = 50%–75% the nucleus colocalized with GR, Q4 = 75%–100% of the nucleus colocalized with GR. Analysis between aged and young groups was performed on both the quartiled and continuous datasets.

To determine size of microglia soma, we isolated the nuclei identified as belonging to microglia cells, then created a three-dimensional dilation mask expanding three pixels beyond the edge of the nucleus. We next transferred the dilation mask to the image of the microglia processes. The volume of microglia expression within the dilation mask was calculated as used as the volume of the soma of the microglia cell. Microglia soma volume was averaged by animal and compared between groups.

### Statistical Analyses

Statistical analyses were performed using SAS 9.4, GraphPad Prism Software and MATLAB 2014a statistical software. The generalized mixed model as well as two-way analysis of variance, non-parametric Kruksal-Wallis, Whitney-Mann U and Kolmogorrov-Smirnov tests were performed to determine significance at *p* value < 0.05 between and among aging and stress conditions. Non-parametric measures were used when a result produced a skewed mean distribution. Pearson correlations were performed on all correlative data presented in this study and all reported *r-values* were created using GraphPad Prism Software. Empirical cumulative distribution functions (eCDFs) graphs were created using Microsoft Excel.

## Results

### Age-Related Changes in Microglial Volume and Immunoreactivity

**Figure 2:**
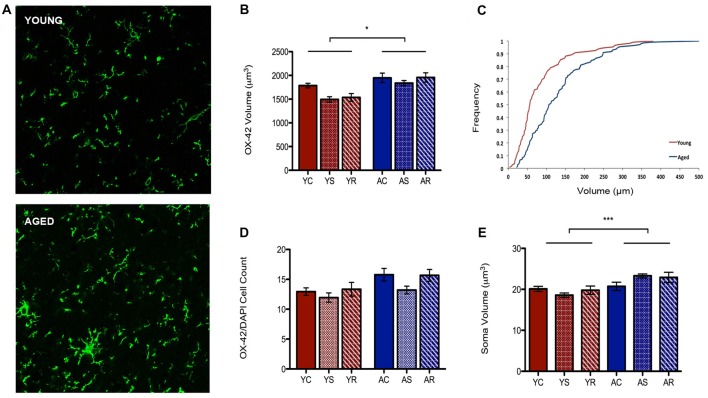
Microglia morphological plasticity during aging. **(A)** Fluorescent images of microglia from the PFC of a young (top) and aged (bottom) rat. **(B)** A significant increase in total microglial volume between young animals and aged animals was observed (*F* = 6.58, *p* = 0.0149); no change was observed across stress conditions (*F* = 0.96, *p* = 0.3941). *p < 0.05. Bar graphs represent the group mean ± SEM. **(C)** Cumulative frequency plot of microglial volume between the young and aged groups. **(D)** Microglia cell number did not differ between the age groups (*F* = 3.30, *p* = 0.0780) or with stress conditions (*F* = 1.29, *p* = 0.2892). Bar graphs represent the group mean ± SEM. **(E)** Soma volume was measured as a morphological indicator of relative immunoreactivity during aging. A significant increase in soma volume between young and aged animals wasobserved (*F* = 15.99, *p* = 0.0003). ***p < 0.001. Bar graphs represent the group mean ± SEM.

To characterize astrocytic morphology in chronic stress and aging, PL sections from control, stressed and stress-recovered young and aged male rats were counter-stained against the microglial marker OX-42 and the astrocytic marker GFAP. DAPI was used for nuclear staining. Additionally, all sections were stained for GRs to determine changes in abundance and cellular localization within each glial subpopulation as described below. A qualitative increase in OX-42 staining in aged compared to young rats was apparent and reflected a significant increase in microglial volume (*F* = 6.58, *p* = 0.0149; Figures 2A,B). Interestingly, we found no effect of stress condition (*F* = 0.96, *p* = 0.3941) or stress-aging interaction (*F* = 0.45, *p* = 0.6390). The age-related shift in microglia volume was confirmed using a cumulative distribution plot, which showed a significant rightward shift towards larger microglial volume with aging (Figure 2C). This volumetric difference was not accompanied by differences in number of OX-42 microglia (*F* = 3.30, *p* = 0.0780; Figure 2D). Microglial number was also not associated with stress conditions (*F* = 1.29, *p* = 0.2892) or interactive aging-stress effects (*F* = 0.17, *p* = 0.8464). In addition, soma size was measured as a morphological indicator of microglial immunoreactivity. A statistically significant increase in soma size was found in aged rats compared to young rats (*F* = 15.99, *p* = 0.0003; Figure 2E).

### Subcellular Age-Related Changes in Glial Glucocorticoid Receptors Are Specific to Microglia

**Figure 3:**
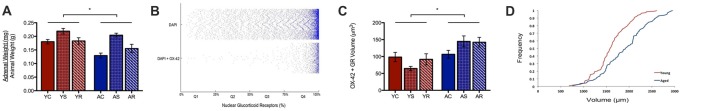
Subcellular analysis of microglial GR. **(A)** Normalized adrenal gland weights of young and aged rats (*U* = 89.0 *p* = 0.01). *p < 0.05. Bar graphs represent the groupmean ± SEM. **(B)** Distributions of nuclear colocalized GRs from all cells (i.e., DAPI) and from microglia (i.e., DAPI +OX-42). The volume of nuclear GRs was plotted as the percent of GR marker within the nucleus and sorted in quartiles for quantitative analysis. The plot shows most cells having greater than 75% GR in the nucleus; in other words, the majority of cells displayed highly dense GR nuclei. **(C)** Non-nuclear microglial GR between young animals and aged animals (*F* = 6.00, *p* = 0.02) and stress conditions (*F* = 0.19, *p* = 0.83). *p < 0.05. Bar graphs represent the group mean ± SEM. **(D)** Cumulative frequency plot of non-nuclear microglial GR volume between young and aged rats.

A possible reason for the observed age-induced microglial volume increase could be the initiation of a chronic inflammatory response in microglia during aging (Sierra et al., 2007, 2008). Our work has previously shown a significant change in adrenal weight across stress conditions (Bloss at al., 2010). Here, the same data shows a significant aging effect, with adrenal weight being lower in aged animals (*U* = 89.0, *p* = 0.01; Figure 3A). To investigate the adrenal response in the PL area, we quantified the nuclear and non-nuclear expression of GR proteins. In microglia, we defined nuclear GR signal as the colocalization of OX-42, DAPI and GR. Astrocytic nuclear analysis was excluded due to limited numbers of GFAP-positive nuclei captured during imaging, which led to an underpowered analysis.

Notably, we found nuclear GR to either be virtually absent or highly dense in the majority of microglia. As can be seen in Figure 3B, the majority of microglial that contain nuclear GRs, have a high content of these receptors. To quantify this, we divided microglia containing nuclear GRs into four quartiles, where Q1 = <25% GR by volume, Q2 = 25%–50% GR by volume, Q3 = 50%–75% GR by volume, Q4 = >75% by volume (Figure 3B). The majority of nuclear GR labeled DAPI + OX-42 expression occupied more than 75% volume in the nucleus of microglia, so further analysis focused on these highly GR dense nuclei. Cell counts were performed on the number of highly GR dense nuclei to determine if their number varied across conditions. However, no significant findings were reported in the aging (*H* = 0.59, *p* = 0.2079), nor stress conditions (*H* = 4.85, *p* = 0.09), and no interactive effects were significant (*H* = 7.15, *p* = 0.21).

In addition to nuclear GR, non-nuclear GR expression was quantified by calculating the total colocalization between GR and OX-42 then, subtracting all GR colocalized with DAPI. We found that aged rats expressed a significantly higher volume of non-nuclear microglial GR as compared to that of young rats (*F* = 6.00, *p* = 0.02; Figure 3C). Again, no significant differences were found among the stress groups (*F* = 0.19, *p* = 0.83) and no interactive effects were observed (*F* = 1.06, *p* = 0.36). A rightward shift in the distribution of non-nuclear microglial GR volume distribution with aging confirmed the increase in density (Figure 3D).

### No Age-Related Changes Observed in Astrocytes

**Figure 4:**
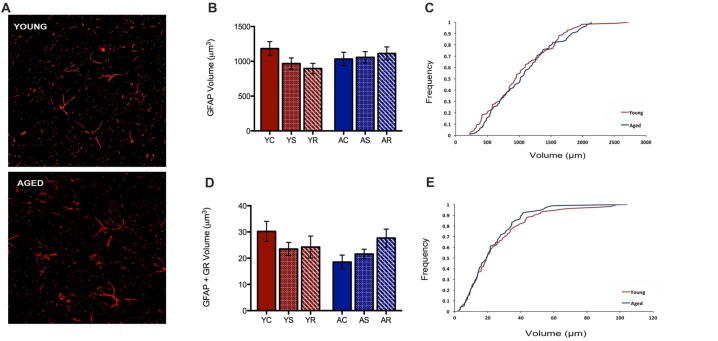
Astrocytes show no change in volume nor non-nuclear GR volume. **(A)** Fluorescent images of astrocytes from the PFC of a young (top) and aged (bottom) rat. **(B)** Total volume in astrocytes across age groups (*F* = 0.36, *p* = 0.5543) and stress conditions (*F* = 0.23, *p* = 0.7929). Bar graphs represent the group mean ± SEM. **(C)** Cumulative frequency plot of astrocytic volume between the young and aged groups. **(D)** Non-nuclear GR volume in astrocytes across ages (*F* = 0.55, *p* = 0.47) and stress condition (*F* = 0.36, *p* = 0.70). Bar graphs represent the group mean ± SEM. **(E)** Cumulative frequency plot of astrocytic GR volume between the young and aged groups.

In the present study, our custom automated analysis failed to accurately capture GFAP/DAPI colocalization and thus, only a low number of GFAP positive nuclei were isolated. Therefore, our analysis was limited to GFAP volume analyses. In contrast to microglial data, GFAP+ astrocytes did not reveal any morphological changes between young and aged rats (Figure 4A). GFAP+ astrocytes had no significant change in volume with aging (*F* = 0.36, *p* = 0.5543), stress condition (*F* = 0.23, *p* = 0.7929) or interaction (*F* = 1.10, *p* = 0.3456; Figure 4B). No significant shift in the distribution of astrocytic volume during aging was observed (Figure 4C). The total non-nuclear GR volume in astrocytes was analyzed using the same methods and no statistically significant results were found across aging (*F* = 0.55, *p* = 0.47) and the stress conditions (*F* = 0.36, *p* = 0.70; Figure 4D). No interactive effects were significant (*F* = 1.57, *p* = 0.22). No significant shift in the distribution of non-nuclear GR volume in astrocytes was observed (Figure 4E).

### Changes in Spine Density Correlate With Increased Microglial Volume and Increased Non-nuclear Glucocorticoid Receptor Expression During Aging

**Figure 5:**
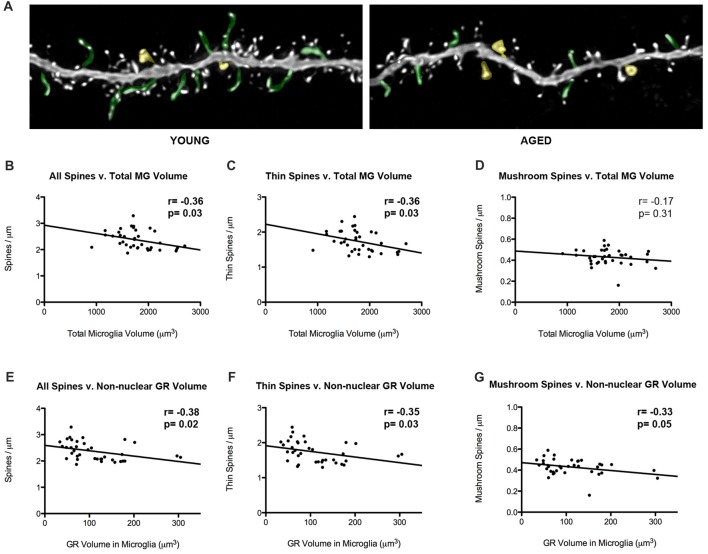
Microglial volumes and expression of non-nuclear microglial GR correlate with prefrontal dendritic spine density. **(A)** Twenty-five micrometer by 10 μm images of spines seen on layer III PL pyramidal dendrites in old and young rat models. Examples of thin spines are highlighted in green, and examples of mushroom spines are highlighted in yellow. **(B)** The relationship between microglia volume and dendritic spine densities from layer III PL PFC pyramidal cells (*r* = –0.36, *p* = 0.03). **(C)** Relationship between thin spines and microglia volume (*r* = –0.36, *p* = 0.03). **(D)** Relationship between mushroom spines and microglia volume (*r* = –0.17, *p* = 0.31). **(E–G)** Same as **(B–D)**, but with non-nuclear microglial GR volume (**E**: *r* = –0.38, *p* = 0.02; **F**; *r* = –0.35, *p* = 0.03; **G**; *r* = –0.33, *p* = 0.05). Significant *r* values with *p* < 0.05 outlined in bold.

Our laboratory has previously showed that thin spine density decreases in aged rats in comparison to young rats (Figure 5A). To look into how glial-synaptic interactions may contribute to differences in synaptic connectivity, we correlated microglia volume and GR expression to the density of dendritic spines on layer III pyramidal cells that were previously reported (Bloss et al., 2011). Interestingly, we found a negative correlation between total volume of microglia and overall spine densities (*r* = –0.36, *p* = 0.03; Figure 5B). This relationship was driven by thin spines, as a negative correlation was found between microglia volume and thin spine density (*r* = –0.36, *p* = 0.03; Figure 5C), but not between microglia volume and mushroom spine density (*r* = –0.17, *p* = 0.31; Figure 5D). A similar significant negative correlation was found between overall spine density and non-nuclear microglial GR volume (*r* = –0.38, *p* = 0.02; Figure 5E). Here, the negative correlation held for both thin (*r* = –0.35, *p* = 0.03; Figure 5F) and mushroom subtypes (*r* = –0.33, *p* = 0.05; Figure 5G).

## Discussion

The conventional view of both astrocytes and microglia is that they provide critical support for the proper function of local neural networks, though recent reports have provided strong evidence that both cell types can also actively contribute to circuit function (Belanger and Magistretti, 2009; Allaman et al., 2011; Araque et al., 2014; Martín et al., 2015). Previous studies have shown that stress and age-related structural plasticity of prefrontal cortical neurons is related to detriments in prefrontal function, though virtually no data are available regarding the morphological plasticity of glial cell types under these same conditions (Bloss et al., 2010, 2011; McEwen and Morrison, 2013).

The present study addressed whether mPFC microglia and astrocytes also exhibited morphological and molecular plasticity in response to aging and stress. Surprisingly, we show age-related changes in morphology and GR protein expression are strongly selective between these two glial cell types, with microglia showing increases in total volume and non-nuclear GR in aged rats. In contrast, neither cell type showed any change in morphology or localization of GRs in response to stress exposure. Interestingly, the selective changes in microglial morphology during aging were associated with reductions in the density of dendritic spines on pyramidal cell dendrites, suggesting common mechanisms may underlie both microglial and neuronal age-related plasticity. Collectively, these data are consistent with the hypothesis that selective changes in the local microglial network co-occur alongside large-scale synaptic remodeling during neocortical aging.

### Selective Age-Related Alterations in PFC Microglial Morphology and Immunoreactivity

Previous reports have suggested that there are functional implications for changes in microglial morphology; for example, microglia serve as resident surveyors within the central nervous system by detecting and responding to changes in the local environment (Conde and Streit, 2006; Tremblay et al., 2011). These cells exist in ramified or active states depending on its function. For instance, under acute injury, microglia will proceed through various active states responding via neuroprotective mechanisms (Felger et al., 2010; Walker et al., 2014). Morphological analyses were also paired with behavioral LPS challenge resulting in a morphological distinction between active and ramified states (Kongsui et al., 2015). Thus, these states can be successfully identified using morphological analyses.

Furthermore, age-related changes in microglial morphology have previously been considered within the context of a general increase in inflammatory tone. Microglial morphology is a reliable predictor of the baseline inflammatory profile in aged mice *ex vivo* (Conde and Streit, 2006; Sierra et al., 2007). In the hippocampus, greater glucocorticoid receptor activation sensitizes microglia in aged rats (Barrientos et al., 2015). In the PFC, morphological analyses revealed increased microglial process length and branch complexity in aged rats (Kongsui et al., 2014). Additionally, many studies have suggested increased soma size as an indicator of microglial activation (Kozlowski and Weimer, 2012; Kongsui et al., 2014; Davis et al., 2017). In light of this, we considered a volumetric soma analysis and found that soma size increased in aged rats compared to young rats. This finding argues an age-related increase in microglial activation and consistent with previous reports of an increased inflammatory profile in aged animals (Sierra et al., 2007). Taken together, we show here microglia in the PFC exhibit altered morphology and immunoreactivity and this trend suggests these age-related changes are selective for microglia and not accompanied by remodeling of astrocytic processes at this level of resolution. Our data do not address whether or not alterations have occurred in astrocytic processes that have been shown to play a role in plasticity at the synaptic level (Perea et al., 2009; Eroglu and Barres, 2010; Gómez-Gonzalo et al., 2017). In addition, it is important to note that our analyses did not include an examination of astrocytic cell count due to a limitation of our custom automated image analysis methods. Since GFAP signal was not uniform within the cell body, we were unable to colocalize it accurately with the DAPI marker. The results revealed an unreliability low number of GFAP positive astrocytic nuclei. Instead, our astrocytic analyses observed non-specific morphological measures of GFAP, revealing astrocytes did not exhibit the same volume changes in morphology or GR expression as microglia during aging. In light of this limitation, our findings cannot offer any additional support for previously observed changes in astrocyte numbers in the PFC and hippocampus as described in other stress paradigms (Czeh et al., 2006; Lucassen et al., 2014).

### Cytoplasmic Microglial Glucocorticoid Receptors Are Affected by Aging

One prominent regulator of central inflammation is the glucocorticoid receptor which can mediate both nuclear and non-nuclear signaling cascades to reprogram the cellular immune response (Limbourg and Liao, 2003; Chinenov et al., 2013). Previous studies have suggested that microglia contribute to the local inflammatory state (Kreutzberg, 1996; Wake et al., 2013; Wu et al., 2015). Although it has been previously shown that nuclear GR upregulation occurs in microglia under neuroinflammatory events *in vitro*, little is known of non-nuclear GR. In PFC, GR is widely expressed in glial cells playing both genomic and non-genomic roles (Tasker et al., 2006; Du et al., 2009; Hill and McEwen, 2009). During aging, glucocorticoids are released at higher baseline and peak levels during the circadian cycle, and stress responses are usually exaggerated (Sapolsky, 1999). Stress exposure or exogenous glucocorticoid exposure produce reduced GR levels and in general these features are recapitulated by age-related increases in glucocorticoids (Peiffer et al., 1991; Mizoguchi et al., 2003).

It is somewhat surprising, then, that we find here elevated levels of non-nuclear GR in aging microglia in the rat PFC. Our results show that while nuclear GR expression remains consistent in microglia, non-nuclear GR expression increases, and thus, reveal an age-related discrepancy. Our finding is particularly interesting since it has now been observed that nuclear GR levels are stable in both neurons and glia as well as in both the hippocampus and PFC (Wang et al., 2013). Thus, it is instead non-nuclear microglial GR, not nuclear GR, that exhibit changes during aging. Along with studies showing increased microglia activation in aged animals, our observation provides a possible theory that non-nuclear GR may play a role in changing or altered inflammatory profiles during aging (Conde and Streit, 2006; Norden and Godbout, 2013).

Beyond the findings of this study, we propose that this may reflect an age-related alteration in GR shuttling to and from the nucleus, resulting in increased nonnuclear GR as a result of dysregulation of the nuclear negative feedback mechanism. A second possible alteration might result from changes in the level of the 11β hydroxysteroid dehydrogenase type I (11bHSD), which is an enzyme that catalyzes inactive CORT to active cortisone and is an important enzyme in local CORT conversion. 11bHSD is largely expressed in activated microglia (Sakai et al., 1992; Gottfried-Blackmore et al., 2010; Wyrwoll et al., 2011); however, little is known of the importance of this enzyme in microglia during aging. Interestingly, while studies have shown an increase in 11bHSD to be detrimental during aging in neurons, our finding shows that adrenal weight decreases in aged rats and thus, suggests that there is either decrease or dysfunction in 11bHSD (Yau et al., 2001, 2011). We hypothesize the latter to be true and that microglia may be in a state of reduced sensitivity to changes in global corticosteroid levels. The probable mechanistic implications for linking this enzyme to non-nuclear GR makes it a suitable target for future studies.

### Chronic Stress Exposure Influences Neuronal Morphology, However, Not Glial

Surprisingly, we did not find any significant changes across microglial or astrocytic analyses among the stress conditions. These results suggest that glial cell morphology may not be impacted by changes associated with chronic restraint stress, and stand in stark contrast to the large scale dendritic and synaptic remodeling that occurs in neighboring pyramidal neurons. Although, our data do not rule out ultrastructural alterations in astrocytic or microglial processes that would affect synaptic function. Moreover, the lack of a stress effect on glial GR levels contrast with data from neurons, as nuclear GR downregulation have been shown to occur consistently during exposure to chronic stress or stress-like levels of exogenous CORT (Sapolsky, 1999; Chiba et al., 2012; Popoli et al., 2012).

### Correlation Between Microglial Changes and Excitatory Connectivity

We took advantage of the previously collected spine data to determine if any significant relationships existed between these glial changes and the age-related changes of layer III pyramidal cell dendritic spines. Total spine densities had a negative correlation with both total microglia volume and non-nuclear GR volume in all animals. In other words, a loss in overall spine density was observed as microglial volume or non-nuclear GR volume increased.

A possible mechanism influencing thin spines may indeed be age-related changes in the microglial network. Our data are consistent with the “synaptic stripping” hypothesis, which has been suggested as a putative mechanistic link between glial activation and spine plasticity (Trapp et al., 2007; Araya et al., 2014; Wu et al., 2015; Hong and Stevens, 2016). Although, our findings do not provide any direct evidence since we do not have synaptic resolution for the microglia and astrocyte analyses. Future studies should explore more conclusive measures at the synaptic level, though our findings are consistent with recent literature emphasizing the role of microglial-neuronal interactions in synaptic pruning and elimination (Stephan et al., 2012; Wake et al., 2013; Schafer and Stevens, 2015; Wu et al., 2015; Weinhard et al., 2018).

### Glucocorticoids, Microglia and the Aging Brain

Our exploration of GR in microglia offers a new perspective in morphological assessment of microglia during aging. As shown here in microglial cells in aged rats, glucocorticoids now have been shown to have effects beyond chronic stress in neuronal cells in the mPFC. As microglial-neuronal interactions continue to play a vital role in proper function of the human brain, this is an important step to understanding the subcellular changes occurring beyond microglial activation (Weinhard et al., 2018). These assessments taken together may help define and differentiate between diseased and senescent cell types as cognitive decline and dementia becomes increasingly relevant to research in normal aging.

## Author Contributions

TEC and JHM designed the research. TEC and EBB performed the research. EBB and WGJ conducted animal testing and handling. TEC analyzed the data. YSG and DD conducted and contributed to the algorithmic and computational analyses. TEC and WL performed statistical analyses. TEC, BSM and JHM wrote the article.

## Conflict of Interest Statement

The authors declare that the research was conducted in the absence of any commercial or financial relationships that could be construed as a potential conflict of interest.

## References

[B1] Allaman I. Bélanger M. Magistretti P. J. (2011). Astrocyte-neuron metabolic relationships: for better and for worse. Trends Neurosci. 34, 76–87. 10.1016/j.tins.2010.12.001 21236501

[B2] Araque A. Carmignoto G. Haydon P. G. Oliet S. H. R. Robitaille R. Volterra A. (2014). Gliotransmitters travel in time and space. Neuron 81, 728–739. 10.1016/j.neuron.2014.02.007 24559669 PMC4107238

[B3] Araya R. Vogels T. P. Yuste R. (2014). Activity-dependent dendritic spine neck changes are correlated with synaptic strength. Proc. Natl. Acad. Sci. U S A 111, E2895–E2904. 10.1073/pnas.1321869111 24982196 PMC4104910

[B4] Barrientos R. M. Thompson V. M. Kitt M. M. Amat J. Hale M. W. Frank M. G.. (2015). Greater glucocorticoid receptor activation in hippocampus of aged rats sensitizes microglia. Neurobiol. Aging 36, 1483–1495. 10.1016/j.neurobiolaging.2014.12.003 25559333 PMC4346455

[B5] Belanger M. Magistretti P. J. (2009). The role of astroglia in neuroprotection. Dialogues Clin. Neurosci. 11, 281–295. 19877496 10.31887/DCNS.2009.11.3/mbelanger PMC3181926

[B6] Bloss E. B. Janssen W. G. McEwen B. S. Morrison J. H. (2010). Interactive effects of stress and aging on structural plasticity in the prefrontal cortex. J. Neurosci. 30, 6726–6731. 10.1523/JNEUROSCI.0759-10.2010 20463234 PMC2888496

[B7] Bloss E. B. Janssen W. G. Ohm D. T. Yuk F. J. Wadsworth S. Saardi K. M.. (2011). Evidence for reduced experience-dependent dendritic spine plasticity in the aging prefrontal cortex. J. Neurosci. 31, 7831–7839. 10.1523/JNEUROSCI.0839-11.2011 21613496 PMC3398699

[B8] Bloss E. B. Puri R. Yuk F. Punsoni M. Hara Y. Janssen W. G.. (2013). Morphological and molecular changes in aging rat prelimbic prefrontal cortical synapses. Neurobiol. Aging 34, 200–210. 10.1016/j.neurobiolaging.2012.05.014 22727942 PMC3483440

[B9] Chiba S. Numakawa T. Ninomiya M. Richards M. C. Wakabayashi C. Kunugi H. (2012). Chronic restraint stress causes anxiety- and depression-like behaviors, downregulates glucocorticoid receptor expression and attenuates glutamate release induced by brain-derived neurotrophic factor in the prefrontal cortex. Prog. Neuropsychopharmacol. Biol. Psychiatry 39, 112–119. 10.1016/j.pnpbp.2012.05.018 22664354

[B10] Chinenov Y. Gupte R. Rogatsky I. (2013). Nuclear receptors in inflammation control: repression by GR and beyond. Mol. Cell. Endocrinol. 380, 55–64. 10.1016/j.mce.2013.04.006 23623868 PMC3787948

[B11] Conde J. R. Streit W. J. (2006). Microglia in the aging brain. J. Neuropathol. Exp. Neurol. 65, 199–203. 10.1097/01.jnen.0000202887.22082.63 16651881

[B12] Cotrina M. L. Nedergaard M. (2002). Astrocytes in the aging brain. J. Neurosci. Res. 67, 1–10. 10.1002/jnr.10121 11754075

[B13] Czeh B. Simon M. Schmelting B. Hiemke C. Fuchs E. (2006). Astroglial plasticity in the hippocampus is affected by chronic psychosocial stress and concomitant fluoxetine treatment. Neuropsychopharmacology 31, 1616–1626. 10.1038/sj.npp.1300982 16395301

[B14] Davis B. M. Salinas-Navarro M. Cordeiro M. F. Moons L. De Groef L. (2017). Characterizing microglia activation: a spatial statistics approach to maximize information extraction. Sci. Rep. 7:1576. 10.1038/s41598-017-01747-8 28484229 PMC5431479

[B15] Diorio D. Viau V. Meaney M. J. (1993). The role of the medial prefrontal cortex (cingulate gyrus) in the regulation of hypothalamic-pituitary-adrenal responses to stress. J. Neurosci. 13, 3839–3847. 10.1523/JNEUROSCI.13-09-03839.1993 8396170 PMC6576467

[B16] Du G. Drexler G. A. Friedland W. Greubel C. Hable V. Krucken R.. (2011). Spatial dynamics of DNA damage response protein foci along the ion trajectory of high-LET particles. Radiat. Res. 176, 706–715. 10.1667/rr2592.1 21797665

[B17] Du J. McEwen B. S. Manji H. K. (2009). Glucocorticoid receptors modulate mitochondrial function. Commun. Integr. Biol. 2, 350–352. 10.4161/cib.2.4.8554 19721888 PMC2734045

[B18] Eroglu C. Barres B. A. (2010). Regulation of synaptic connectivity by glia. Nature 468, 223–231. 10.1038/nature09612 21068831 PMC4431554

[B19] Felger J. C. Abe T. Kaunzner U. W. Gottfried-Blackmore A. Gal-Toth J. McEwen B. S.. (2010). Brain dendritic cells in ischemic stroke: time course, activation state, and origin. Brain Behav. Immun. 24, 724–737. 10.1016/j.bbi.2009.11.002 19914372 PMC2885548

[B20] Frank M. G. Thompson B. M. Watkins L. R. Maier S. F. (2012). Glucocorticoids mediate stress-induced priming of microglial pro-inflammatory responses. Brain Behav. Immun. 26, 337–345. 10.1016/j.bbi.2011.10.005 22041296 PMC5652300

[B21] Givalois L. (2014). The glucocorticoid receptors regulation in Alzheimer’s disease. Neurobiol. Aging 35, e17–e18. 10.1016/j.neurobiolaging.2013.12.012 24411291

[B22] Gómez-Gonzalo M. Martin-Fernandez M. Martínez-Murillo R. Mederos S. Hernández-Vivanco A. Jamison S.. (2017). Neuron-astrocyte signaling is preserved in the aging brain. Glia 65, 569–580. 10.1002/glia.23112 28130845 PMC5314210

[B23] Gottfried-Blackmore A. Sierra A. McEwen B. S. Ge R. Bulloch K. (2010). Microglia express functional 11 β-hydroxysteroid dehydrogenase type 1. Glia 58, 1257–1266. 10.1002/glia.21007 20544861

[B24] Hill M. N. McEwen B. S. (2009). Endocannabinoids: the silent partner of glucocorticoids in the synapse. Proc. Natl. Acad. Sci. U S A 106, 4579–4580. 10.1073/pnas.0901519106 19293387 PMC2660761

[B25] Hong S. Stevens B. (2016). Microglia: phagocytosing to clear, sculpt, and eliminate. Dev. Cell 38, 126–128. 10.1016/j.devcel.2016.07.006 27459063

[B26] Kettenmann H. Kirchhoff F. Verkhratsky A. (2013). Microglia: new roles for the synaptic stripper. Neuron 77, 10–18. 10.1016/j.neuron.2012.12.023 23312512

[B27] Kongsui R. Beynon S. B. Johnson S. J. Walker F. R. (2014). Quantitative assessment of microglial morphology and density reveals remarkable consistency in the distribution and morphology of cells within the healthy prefrontal cortex of the rat. J. Neuroinflammation 11:182. 10.1186/s12974-014-0182-7 25343964 PMC4213482

[B28] Kongsui R. Johnson S. J. Graham B. A. Nilsson M. Walker F. R. (2015). A combined cumulative threshold spectra and digital reconstruction analysis reveal structural alterations of microglia within the prefrontal cortex following low-dose LPS administration. Neuroscience 310, 629–640. 10.1016/j.neuroscience.2015.09.061 26440295

[B29] Kozlowski C. Weimer R. M. (2012). An automated method to quantify microglia morphology and application to monitor activation state longitudinally *in vivo*. PLoS One 7:e31814. 10.1371/journal.pone.0031814 22457705 PMC3294422

[B30] Kreutzberg G. W. (1996). Microglia: a sensor for pathological events in the CNS. Trends Neurosci. 19, 312–318. 10.1016/0166-2236(96)10049-7 8843599

[B31] Landfield P. W. Blalock E. M. Chen K. C. Porter N. M. (2007). A new glucocorticoid hypothesis of brain aging: implications for Alzheimer’s disease. Curr. Alzheimer Res. 4, 205–212. 10.2174/156720507780362083 17430248 PMC3573879

[B32] Limbourg F. P. Liao J. K. (2003). Nontranscriptional actions of the glucocorticoid receptor. J. Mol. Med. 81, 168–174. 10.1007/s00109-003-0418-y 12682725 PMC2649714

[B33] Lucassen P. J. Pruessner J. Sousa N. Almeida O. F. Van Dam A. M. Rajkowska G.. (2014). Neuropathology of stress. Acta Neuropathol. 127, 109–135. 10.1007/s00401-013-1223-5 24318124 PMC3889685

[B34] Martín R. Bajo-Grañeras R. Moratalla R. Perea G. Araque A. (2015). Circuit-specific signaling in astrocyte-neuron networks in basal ganglia pathways. Science 349, 730–734. 10.1126/science.aaa7945 26273054

[B35] McEwen B. S. (2006). Protective and damaging effects of stress mediators: central role of the brain. Dialogues Clin. Neurosci. 8, 367–381. 17290796 10.31887/DCNS.2006.8.4/bmcewen PMC3181832

[B36] McEwen B. S. Morrison J. H. (2013). The brain on stress: vulnerability and plasticity of the prefrontal cortex over the life course. Neuron 79, 16–29. 10.1016/j.neuron.2013.06.028 23849196 PMC3753223

[B37] Mika A. Mazur G. J. Hoffman A. N. Talboom J. S. Bimonte-Nelson H. A. Sanabria F.. (2012). Chronic stress impairs prefrontal cortex-dependent response inhibition and spatial working memory. Behav. Neurosci. 126, 605–619. 10.1037/a0029642 22905921 PMC3463780

[B38] Mizoguchi K. Ishige A. Aburada M. Tabira T. (2003). Chronic stress attenuates glucocorticoid negative feedback: involvement of the prefrontal cortex and hippocampus. Neuroscience 119, 887–897. 10.1016/s0306-4522(03)00105-2 12809708

[B39] Norden D. M. Godbout J. P. (2013). Microglia of the aged brain: primed to be activated and resistant to regulation. Neuropathol. Appl. Neurobiol. 39, 19–34. 10.1111/j.1365-2990.2012.01306.x 23039106 PMC3553257

[B40] O’Callaghan J. Brinton R. McEwen B. S. (1991). Glucocorticoids regulate the synthesis of glial fibrillary acidic protein in intact and adrenalectomized rats but do not affect its expression following brain injury. J. Neurochem. 57, 860–869. 10.1111/j.1471-4159.1991.tb08230.x 1677678

[B41] Peiffer A. Barden N. Meaney M. J. (1991). Age-related changes in glucocorticoid receptor binding and mRNA levels in the rat brain and pituitary. Neurobiol. Aging 12, 475–479. 10.1016/0197-4580(91)90076-v 1770983

[B42] Perea G. Navarrete M. Araque A. (2009). Tripartite synapses: astrocytes process and control synaptic information. Trends Neurosci. 32, 421–431. 10.1016/j.tins.2009.05.001 19615761

[B43] Popoli M. Yan Z. McEwen B. S. Sanacora G. (2012). The stressed synapse: the impact of stress and glucocorticoids on glutamate transmission. Nat. Rev. Neurosci. 13, 22–37. 10.1038/nrn3138 22127301 PMC3645314

[B44] Rhen T. Cidlowski J. A. (2005). Antiinflammatory action of glucocorticoids—new mechanisms for old drugs. N. Engl. J. Med. 353, 1711–1723. 10.1056/nejmra050541 16236742

[B45] Ros-Bernal F. Hunot S. Herrero M. T. Parnadeau S. Corvol J. C. Lu L.. (2011). Microglial glucocorticoid receptors play a pivotal role in regulating dopaminergic neurodegeneration in parkinsonism. Proc. Natl. Acad. Sci. U S A 108, 6632–6637. 10.1073/pnas.1017820108 21467220 PMC3080980

[B46] Sakai R. R. Lakshmi V. Monder C. McEwen B. S. (1992). Immunocytochemical localization of 11 β-hydroxysteroid dehydrogenase in hippocampus and other brain regions of the rat. J. Neuroendocrinol. 4, 101–106. 10.1111/j.1365-2826.1992.tb00351.x 21554583

[B47] Sapolsky R. M. (1999). Glucocorticoids, stress, and their adverse neurological effects: relevance to aging. Exp. Gerontol. 34, 721–732. 10.1016/s0531-5565(99)00047-9 10579633

[B48] Schafer D. P. Stevens B. (2015). Microglia function in central nervous system development and plasticity. Cold Spring Harb. Perspect. Biol. 7:a020545. 10.1101/cshperspect.a020545 26187728 PMC4588063

[B49] Schindelin J. Arganda-Carreras I. Frise E. Kaynig V. Longair M. Pietzsch T.. (2012). Fiji: an open-source platform for biological-image analysis. Nat. Methods 9, 676–682. 10.1038/nmeth.2019 22743772 PMC3855844

[B50] Shih A. Y. Fernandes H. B. Choi F. Y. Kozoriz M. G. Liu Y. Li P.. (2006). Policing the police: astrocytes modulate microglial activation. J. Neurosci. 26, 3887–3888. 10.1523/JNEUROSCI.0936-06.2006 16611803 PMC6673885

[B52] Sierra A. Gottfried-Blackmore A. C. McEwen B. S. Bulloch K. (2007). Microglia derived from aging mice exhibit an altered inflammatory profile. Glia 55, 412–424. 10.1002/glia.20468 17203473

[B51] Sierra A. Gottfried-Blackmore A. Milner T. A. McEwen B. S. Bulloch K. (2008). Steroid hormone receptor expression and function in microglia. Glia 56, 659–674. 10.1002/glia.20644 18286612

[B53] Sinclair D. Fillman S. G. Webster M. J. Weickert C. S. (2013). Dysregulation of glucocorticoid receptor co-factors FKBP5, BAG1 and PTGES3 in prefrontal cortex in psychotic illness. Sci. Rep. 3:3539. 10.1038/srep03539 24345775 PMC3866598

[B54] Stephan A. H. Barres B. A. Stevens B. (2012). The complement system: an unexpected role in synaptic pruning during development and disease. Annu. Rev. Neurosci. 35, 369–389. 10.1146/annurev-neuro-061010-113810 22715882

[B55] Tasker J. G. Di S. Malcher-Lopes R. (2006). Minireview: rapid glucocorticoid signaling via membrane-associated receptors. Endocrinology 147, 5549–5556. 10.1210/en.2006-0981 16946006 PMC3280589

[B56] Trapp B. D. Wujek J. R. Criste G. A. Jalabi W. Yin X. Kidd G. J.. (2007). Evidence for synaptic stripping by cortical microglia. Glia 55, 360–368. 10.1002/glia.20462 17136771

[B57] Treccani G. Musazzi L. Perego C. Milanese M. Nava N. Bonifacino T.. (2014). Stress and corticosterone increase the readily releasable pool of glutamate vesicles in synaptic terminals of prefrontal and frontal cortex. Mol. Psychiatry 19, 433–443. 10.1038/mp.2014.5 24535456

[B58] Tremblay M. È. Stevens B. Sierra A. Wake H. Bessis A. Nimmerjahn A. (2011). The role of microglia in the healthy brain. J. Neurosci. 31, 16064–16069. 10.1523/JNEUROSCI.4158-11.2011 22072657 PMC6633221

[B59] van Steensel B. Brink M. van der Meulen K. van Binnendijk E. P. Wansink D. G. de Jong L.. (1995). Localization of the glucocorticoid receptor in discrete clusters in the cell nucleus. J. Cell Sci. 108, 3003–3011. 8537440 10.1242/jcs.108.9.3003

[B60] Vertes R. P. (2004). Differential projections of the infralimbic and prelimbic cortex in the rat. Synapse 51, 32–58. 10.1002/syn.10279 14579424

[B61] Wake H. Moorhouse A. J. Miyamoto A. Nabekura J. (2013). Microglia: actively surveying and shaping neuronal circuit structure and function. Trends Neurosci. 36, 209–217. 10.1016/j.tins.2012.11.007 23260014

[B62] Walker F. R. Beynon S. B. Jones K. A. Zhao Z. Kongsui R. Cairns M.. (2014). Dynamic structural remodelling of microglia in health and disease: a review of the models, the signals and the mechanisms. Brain Behav. Immun. 37, 1–14. 10.1016/j.bbi.2013.12.010 24412599

[B63] Wang Q. Van Heerikhuize J. Aronica E. Kawata M. Seress L. Joels M.. (2013). Glucocorticoid receptor protein expression in human hippocampus; stability with age. Neurobiol. Aging 34, 1662–1673. 10.1016/j.neurobiolaging.2012.11.019 23290588

[B64] Weinhard L. di Bartolomei G. Bolasco G. Machado P. Schieber N. L. Neniskyte U.. (2018). Microglia remodel synapses by presynaptic trogocytosis and spine head filopodia induction. Nat. Commun. 9:1228. 10.1038/s41467-018-03566-5 29581545 PMC5964317

[B65] Wu Y. Dissing-Olesen L. MacVicar B. A. Stevens B. (2015). Microglia: dynamic mediators of synapse development and plasticity. Trends Immunol. 36, 605–613. 10.1016/j.it.2015.08.008 26431938 PMC4841266

[B66] Wyrwoll C. S. Holmes M. C. Seckl J. R. (2011). 11β-hydroxysteroid dehydrogenases and the brain: from zero to hero, a decade of progress. Front. Neuroendocrinol. 32, 265–286. 10.1016/j.yfrne.2010.12.001 21144857 PMC3149101

[B67] Yau J. L. Noble J. Kenyon C. J. Hibberd C. Kotelevtsev Y. Mullins J. J.. (2001). Lack of tissue glucocorticoid reactivation in 11β -hydroxysteroid dehydrogenase type 1 knockout mice ameliorates age-related learning impairments. Proc. Natl. Acad. Sci. U S A 98, 4716–4721. 10.1073/pnas.071562698 11274359 PMC31900

[B68] Yau J. L. Noble J. Seckl J. R. (2011). 11β-hydroxysteroid dehydrogenase type 1 deficiency prevents memory deficits with aging by switching from glucocorticoid receptor to mineralocorticoid receptor-mediated cognitive control. J. Neurosci. 31, 4188–4193. 10.1523/JNEUROSCI.6145-10.2011 21411659 PMC3132450

[B69] Yu S. Yang S. Holsboer F. Sousa N. Almeida O. F. (2011). Glucocorticoid regulation of astrocytic fate and function. PLoS One 6:e22419. 10.1371/journal.pone.0022419 21811605 PMC3141054

